# Assessment of Padding Elements Wear of Belt Conveyors Working in Combination of Rubber–Quartz–Metal Condition

**DOI:** 10.3390/ma14154323

**Published:** 2021-08-02

**Authors:** Dawid Romek, Dariusz Ulbrich, Jaroslaw Selech, Jakub Kowalczyk, Roksana Wlad

**Affiliations:** Department of Transport and Civil Engineering, Institute of Machines and Motor Vehicles, 60-965 Poznan, Poland; dawid.romek@put.poznan.pl (D.R.); jaroslaw.selech@put.poznan.pl (J.S.); jakub.kowalczyk@put.poznan.pl (J.K.); roksana.wlad@student.put.poznan.pl (R.W.)

**Keywords:** belt conveyor, wear, padding

## Abstract

Elements of belt conveyors, like other machine parts, are subject to wear processes. The conveyors transporting the spoil in the quartz sand mine are exposed to accelerated wear due to the effect of quartz on metal elements. Intensive wear of metal parts leads to downtime and the need to replace damage parts which generates additional costs. Therefore, it is important to perform surface treatment of metal elements, which will allow to extend the operation time of belt conveyors by reducing wear. The main objective of the article is to determine the impact of the pad welding process of the surface layer of metal elements on the abrasive wear of elements working in the metal–quartz sand–rubber conditions used in belt conveyors. In this research study, three different types of electrodes were used for pad welding the surface. The wear results obtained on the test stand were compared to wear of the basic element without surface treatment. The average wear value of the samples padded with electrode 3 was about 25% lower than the samples without surface treatment. The main mechanism of sample wear was the abrasion process due to the interaction between the steel surface and hard sand particles. The results presented in the article are important not only for belt conveyor elements but also for other machine parts where it is desirable to reduce abrasive wear.

## 1. Introduction

Machine elements used for the transport of spoil, mined in quartz sand mines, are particularly exposed to abrasive wear during operation [1,2]. The metal–quartz–rubber frictional coupling significantly affects the elements of the conveyors, which must have slight deviations in shape and dimension during their utilization [3]. Otherwise, excessive wear of one of the cooperating elements damages the conveyors, which leads to downtime at work. The research presented in the articles [4,5] on the wear of bentonite sand elements showed that belt conveyors into which the layer of molding sands enters between the belt and the steel element are subject to rapid wear as a result of friction. The shape change caused by friction is responsible for about 85% of element replacements [6] and weakens the structure and causes its malfunctions [7,8,9,10].

One of the methods of surface regeneration is pad welding, especially for elements that are exposed to intensive wear [11,12,13,14,15]. Pad welding is a process that significantly increases the abrasive wear resistance [16,17,18]. The technological operation of applying surface layers of specific properties (i.e., increasing resistance to abrasive wear) that are associated with the basic material results in better durability of the machine elements [18]. The basic types of methods for changing resistance to abrasive wear are electrode pad welding or laser treatment [19]. The first type of surface treatment requires the use of appropriate electrodes that allow the formation of a layer that increases the wear resistance of the elements. In the case of changing the wear resistance of the surface layer of machine parts, there are two types of pad welding [18]:

- Gas,
- Electric arc.

The purpose of such an operation is to make the surface of the element required hardness, which should cause a longer life period. Pad welding is one of the types of regeneration of mechanical parts or shaping the properties of the surface layer before its exploitation. Addition of a layer with increased anticorrosion, abrasive, erosive, and cavitation properties significantly affects the wear of the cooperating parts during the machine operation, especially in difficult working conditions [20].

The wear mechanisms of different materials, which are adhesion, abrasion, fatigue, impact, electrical wear, and chemical wear are described in many publications [21,22,23,24,25,26]. In the literature, two-body and three-body modes of abrasive wear are presented [27]. These two modes of wear are similar. However, the main differences were described in [28] and include time of wear process (three-body abrasive wear is ten times slower than two-body wear mode). In the case of sample–abrasive material–belt of the conveyor belt, the hardness of the surface of the steel element is important. Modifying the surface layer by adding thermally applied coatings (including pad welding) causes changes in the metallographic structure of the material and the diffusion of alloying elements. This process changes the hardness of the working elements such that the surface of the elements has a higher hardness than the abrasive element. The effect of thermal separation and its influence on the structure additionally influences the hardening of the surface and the positive values of resistance to abrasive wear.

The main purpose of the research described in the article is to reduce the wear of steel elements working in the metal–quartz sand–rubber combination, which are used in belt conveyors by using the pad welding process. An additional goal is to verify which type of abrasive wear is dominant in tested combination of materials. Steel elements of belt conveyors operating in mines are exposed to accelerated wear due to the influence of sand particles. This causes frequent damage, downtime, and an increase in the operating costs of the belt conveyors. Therefore, cheap methods of modifying the surface of steel elements are sought, which will reduce wear and extend the lifetime of the conveyor. In the tests performed on a laboratory stand which reproduces the working conditions of steel parts of the conveyors, padding weld process with three different electrodes (different chemical composition) was used. This will allow to determine the optimal treatment of the surface layer in terms of wear resistance. Downtime caused by excessive wear of belt conveyor elements generate significant costs for mines. Therefore, it is important to develop a method of extending the utilization time of these machines, especially taking their difficult operating conditions into account.

## 2. Test Stand and Test Conditions

**Figure 1:**
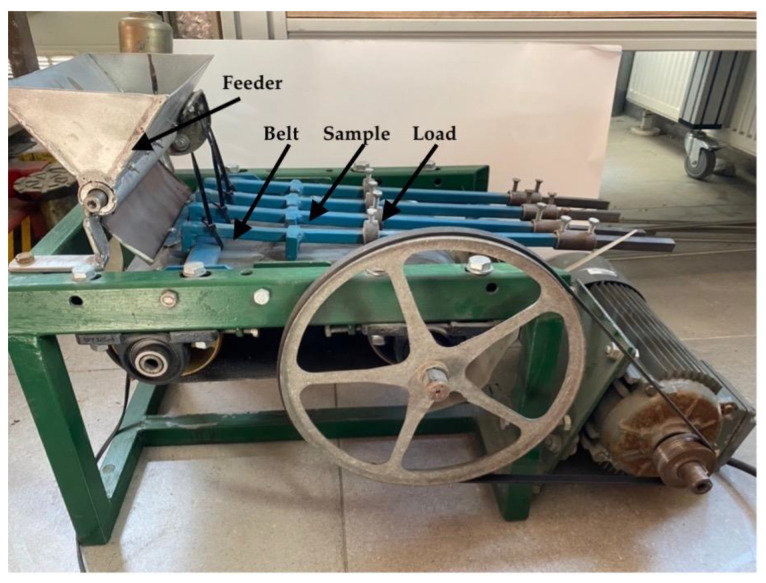
View of the test stand for testing wear of belt conveyor elements made of steel.

The tests used a device that reflects the operating conditions of steel elements of belt conveyors (Figure 1). The machine consists of a transmission belt made of a rubber–textile belt marked EP 630/3, which complies with the PN-EN ISO 14890:2012-06 standard (requirements for the use of conveyor belts) [29]. The tape used for the countersample has a strength of 630 N/mm [30]. The tape processing was performed in accordance with the guidelines contained in [31]. In order to adapt the tape to the test stand, it was shortened and joined with an overlap [32,33] and the elements were joined with a polychloropene adhesive with the designation by CX manufacturer [34,35].

The test stand was designed and manufactured to reflect the actual work of industrial conveyors. The device using a tank with a feeder dispenses the right amount of sand onto the belt. The abrasive is transported to the working elements (samples), which are pressed against the belt by system of levers. The test samples have been profiled in such a way that the quartz sand does not stick to its forehead but is moved over the entire working surface of the sample. The test device shown in Figure 1 is a stand that reflects the working conditions of belt conveyors used in industrial conditions—i.e., in quartz mines. The main difference between the test stand and the industrial conveyor is its dimensions and the possibility of measuring and recording parameters in laboratory conditions. The work parameters of the station can be easy controlled in a wide range such that the different conditions in which the conveyor elements are worn are reflected.

**Figure 2:**
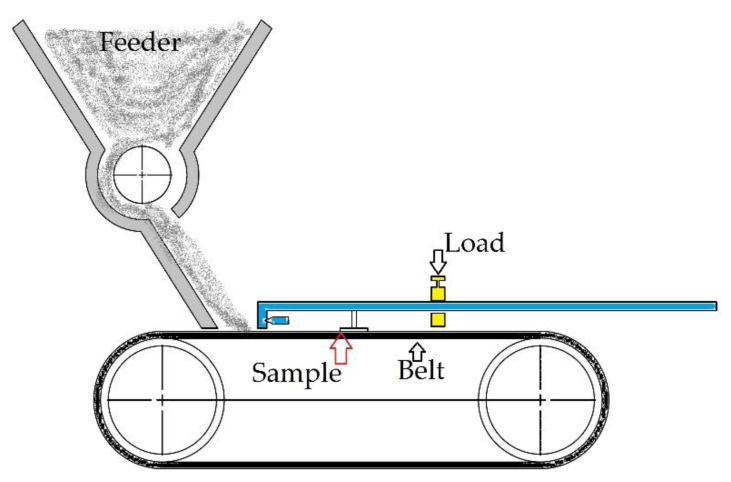
Diagram showing the concept of the test stand.

The system has a sand feeder that feeds the abrasive agent to the conveyor belt. The conveyor belt is driven by a 0.75 kW electric motor. The conveyor belt is 900 mm long and 250 mm wide. The samples were placed on the tape together with the pressure element. An arm system with a weight of 0.1 kg was used as a pressure element. The pressure can be changed by means of the distance of the weight from the actuator, which is the sample. The diagram of the test stand is shown in Figure 2.

**Figure 3:**
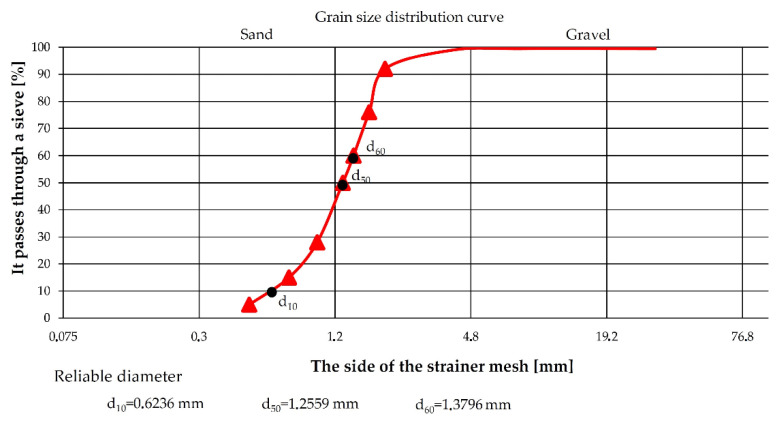
Grain size distribution curve.

Quartz sand with a grain size of 0.8–2.0 mm was used as an abrasive medium and with the particle size distribution curve shown in Figure 3. The *x*-axis shows the mesh diameter through which the tested sand passes or is retained. The *y*-axis shows the percentage of grains of a given diameter that pass through the meshes of a sieve. The parameters d_10_, d_50_, and d_60_ represent the particle diameter, which, together with smaller ones, make up 10%, 50%, and 60% of the fraction.

**Figure 4:**
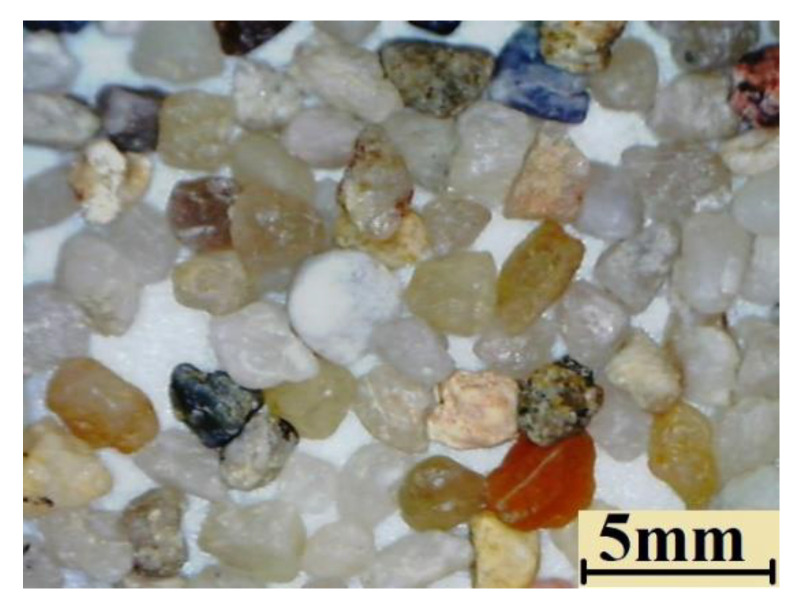
The shape of the abrasive.

The abrasive material used in the laboratory tests complies with the ASTM G65 standard. The shape of the grains of abrasive is shown in Figure 4.

The test sand consists of various chemical particles. The tests were carried out on quartz sand in accordance with the ASTM G65 standard to ensure the repeatability of the tests for all samples. The ASTM G65 standard defines the abrasive material used in tribological testing and was used by other researchers [36]. The exact physicochemical list of particles is presented in Table 1. Sand humidity during the tests was controlled and amounted to 0.2% and its density was equal to 1.5 g/cm^3^. Table 2 shows the remains of the sieve test performed for research purposes.

The tests were conducted in accordance with the parameters set on the test stand presented in Table 3.

## 3. Samples

**Figure 5:**
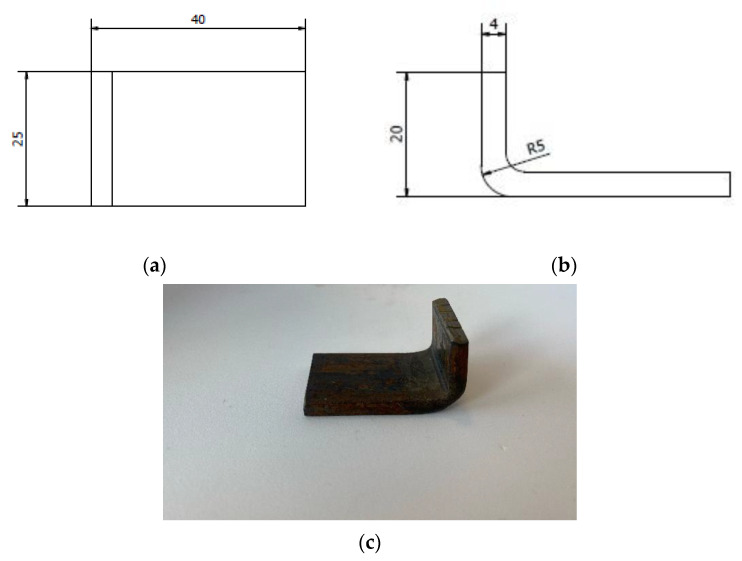
Sample used during the test; dimensions (**a**,**b**) and view (**c**).

The samples were made in accordance with the dimensions shown in Figure 5. The shape of the sample was selected in such a way that the smaller edge was the edge of the pressure of the abrasive mass on the element. This was to prevent the flow of abrasive material over the side walls of the element. In addition, the fillet in the lower part of the element has been profiled in a way that makes it easier for the abrasive mass to get under the element and its flow over the welded surface. For the base material, as well as for three different electrodes used in the pad welding process, five samples were prepared (the total research sample was 20 samples). The specimens were suitably cut from the sheet of cold-rolled material by high-energy water cutting to avoid structural changes in the material and then were formed appropriately by cold working.

S235 steel was used for the tests as a basic steel. This steel was chosen because of its availability and its widespread use in conveyor belts used in industry. The steel composition described by the manufacturer is shown in Table 4.

**Figure 6:**
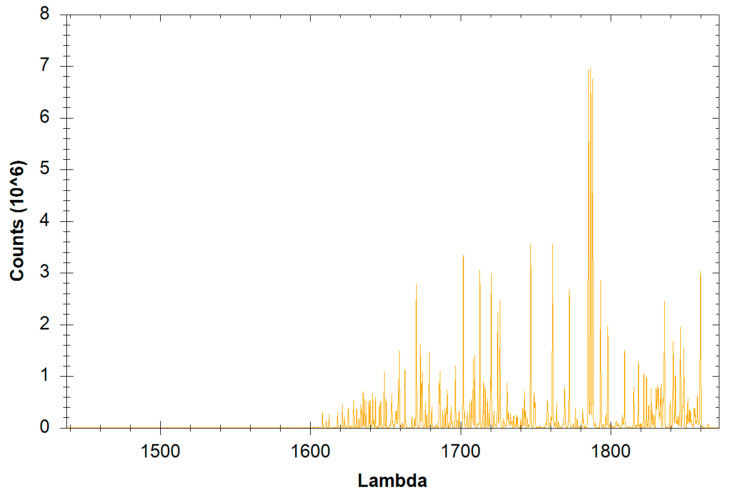
Spectral diagram for steel S235.

The spectral diagram for one sample used during the test was presented in Figure 6. The comparison of the values of the elements contained in S235 steel according to the manufacturer’s data and for the samples used in the tests allows to state that differences in the chemical composition were noted. Nevertheless, the values of individual elements contained in the samples were similar and did not significantly affect the obtained wear results.

In the next stage of the research, three types of electrodes were used for pad welding the surface of the samples with the MMA method (manual arc welding). MMA pad welding process is a method with the use of a coated electrode and an inventory welding machine. In the MMA method, an electric arc is formed and feeding the melting material (metallic core) in a cover that maintains the electric arc in a stable position. These electrodes are characterized by the appropriate content of chromium and molybdenum, which increases the resistance to abrasive wear. The technology of applying layers that increase resistance to abrasion is possible to be used at home with the use of standard inventory welding machines. The chemical composition of the electrodes according to the manufacturer’s data is presented in Table 5. Chemical composition tests were also carried out for the electrodes, verifying the amounts of individual elements. These results are presented in Table 6. Again, deviations in the values of individual elements were noted in relation to the data provided by the manufacturers of the electrodes used in this research.

**Figure 7:**
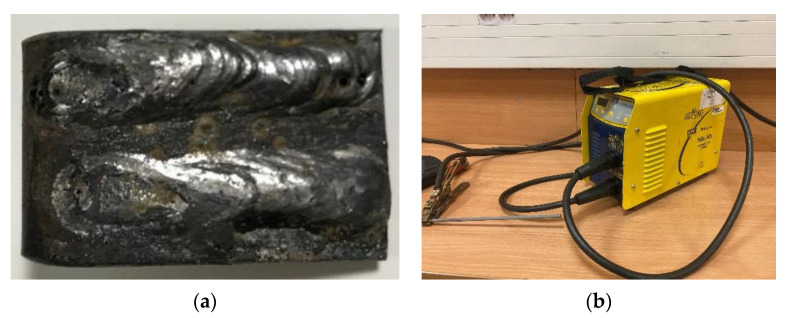
Padding weld process: (**a**) view and shape of the element with padding weld, (**b**) pad welding device with an electrode.

The padding welds were applied to the element in the form of stripes (Figure 7a). The shape of the padding weld was conditioned by the direction of pressure of the abrasive mass and the research of the authors of the work [40]. The pad welding process was carried out using a GYS mi165 arc welder (Figure 7b).

In order to determine the type of structures resulting from the pad welding process of steel samples, microstructure tests of these elements were performed. In terms of structure, the padding weld, weld interface, and base material were analyzed. View of the structures and their description are presented in Table 7, Table 8, Table 9 and Table 10. Preparation of metallographic specimens consisted of cutting out fragments of material with a circular saw. In order not to change the structure of the material, a coolant was used, which allowed to limit the cutting temperature to 120 Celsius degrees. Then, the samples were ground on a Buehler AutoMet 250 automatic clamping grinder–polisher (Buehler, Lake Bluff, IL, USA). The surface of the samples was next etched with the Kalling etchant reagent in order to emphasize the metallographic structure. The samples after production were tested on the Wilson VH3300 device (Buehler, Lake Bluff, IL, USA) to evaluate the hardness distributions in different zones. Metallographic specimens were made in the core of the base material, then on the padding weld and in the weld interface.

As the wear resistance of steel elements overlaid is dependent on the surface hardness, the samples were subjected to hardness tests using the HV_0.1_ method. The results of these measurements for the base sample (without treatment) and the three electrodes used (including the padding weld material and weld interface) are summarized in Table 11.

## 4. Result and Discussion

The samples were thoroughly cleaned in an ultrasonic cleaner, dried at a temperature of 100 °C and weighed before mounting process of the sample on the test stand occurred. Identical procedure was carried out after the tribological test on the test stand. As a result of this test, a weight loss was obtained for individual samples and the averaged results together with the statistical analysis are summarized in Table 12.

Based on the data contained in the table above, it should be stated that the sample without the padding weld was exposed to the highest average wear. The sample with the padding weld made of electrode no. 3 has the lowest average wear. This sample was characterized by the highest hardness value. Similar test results were obtained by the authors of [41], who also observed an increase in abrasive wear resistance in elements with increased hardness of the surface layer. The analysis of the above tests also showed that the growth of the elements which reduce the wear resistance, such as chromium or manganese did not affect the obtained value of the wear of the samples during the tests on the laboratory stand. However, the hardness of the padding weld was the key parameter in terms of the wear resistance of the steel elements of the conveyor belts.

In order to better visualize the test results and to understand the wear mechanisms occurring on the surface of the samples, a scan of the sample surface was performed with the use of an electron scanner—Bruker Alicona InfiniteFocus G5—(Table 13, Brucker Alicona, Graz, Austria) along with the measurements of the basic parameters of the roughness profile (Table 14). In addition, the condition of the top layer was assessed at 20 times magnification under a microscope (Table 15). All of the above tests were performed both before and after the sample wear test.

Examination of the surface of the elements showed that in the case of a material with lower hardness deeper cracks appear and their profile is longer. However, in the case of steel with the highest level of hardness, the penetration of sand into the material was smaller. According to the hardness analysis of the elements, it can be concluded that as the hardness increases, the peak line is sheared and the surface structure is smoothed. This is due to the soft particles that arise during the surfacing in the upper part of the padding weld, and which are initially smoothed by friction. It can also be seen that with the increase in hardness, there is a smaller impact of the abrasive material on the working element, because it results in a higher resistance to abrasive wear. Hardness and roughness analysis as well as tribological tests showed that the increase in hardness clearly affects the abrasive wear.

Analyzing the wear resistance of materials and elements used for quartz belt conveyors the surface of the top layer of samples was tested. The purpose of this study was to verify which type of abrasive wear is dominant. According to the researchers [42], there are three basic types of changes resulting from friction on the surface layer. The basic changes taking place in the surface layer are microscratching, microcutting and microrubbing. The relationship between these types depends on the depth of the furrow and the reference level. Moreover, taking the results presented by the authors into account [43], it is possible to estimate the dominant type of wear and analyze the external surface of the working elements. In the case of samples covered with padding, all three types of wear were noticed. However, the dominant type of wear was the microcutting process. The hardness of surface layer of tested elements has a direct impact on the abrasive wear resistance mechanism. The wear profile of the elements indicates which treatment is characterized by larger or smaller recesses caused by the impact of the abrasive mass stream on the working elements. In the case of lower resistance to abrasive wear, the resulting furrows are larger and more frequent than in the case of using treatment with increased resistance to abrasive wear. The base material is characterized by a large number of cavities created as a result of the pressure of the abrasive mass on the element, while in the case of padded elements, their number and depth are smaller. Taking into account the basic parameters of the surface roughness profile R_a_ and R_z_, it should be stated that for the sample that was not subjected to the pad welding process, the differences in the values before and after the wear test are insignificant. The mean value of the R_a_ parameter decreased from 1.02 to the value of 0.7, while, in the case of the R_z_ parameter, there was a decrease of approximately 30% from the value of 6.79 to the value of 4.48. When analyzing the results of these parameters for the samples that were subjected to the pad welding process, much larger (several times) changes were found. For samples padded with electrode 3, the wear of which was the lowest, the R_z_ parameter value decreased by more than 8 times and the R_a_ parameter value was at the level of 0.34 after the test on the stand (14-fold decrease in value). The roughness of the surface (roughness profile irregularities) formed in the pad welding process was removed by the microcutting process. The above results suggest that the padding weld surface was smoothed during sand pressure due to the wear mechanism (abrasion). The S_ku_ coefficient of the topography height distribution (ordinates) of the surface for the sample not subjected to the pad welding process decreased several times (from 23.4 to 6.91). This is due to the lack of strengthening of the structure due to the precipitation of alloying elements that reduce abrasive wear. This process occurs in the remaining samples that the surface was pad welded. The soft outer layer of the padding, especially surface irregularities, is removed by the abrasion process. The precipitation of elements increases the hardness of the padding weld and increases the abrasive wear resistance of whole surface element. Based on the results of the surface roughness profile and its topography, it can be concluded that the abrasive wear could occur according to the three-body modes [28,44]. Molnar et al. [36], while testing the conveyor belts, found that during the ASTM G65M test, a three-body abrasion mechanism based on the fatigue occurs. For this wear model, the hardness of the surface affected by the abrasive particles is important. The article takes only the results of the wear of steel elements into account. The wear of the rubber conveyor belt has not been taken into account.

## 5. Conclusions

The article includes both wear tests as well as an assessment of the condition of the surface layer of steel elements of belt conveyors given to the pad welding process. These types of parts working in quartz mining conditions are exposed to intense wear. Therefore, it is important to study the effect of various surface treatments on reducing this wear and increasing the utilization time of the machine between repairs. The obtained test results allow us to conclude that the application of the padding weld process reduces wear even by about 25% in relation to parts that have not been subjected to any surface treatment.

Further research directions should include both the use of electrodes with a different chemical composition for the padding weld process of the surface layer, as well as use of more advanced methods of changing the properties of the surface layer, such as laser treatment.

## Figures and Tables

**Table 1:** Chemical composition of the abrasive [37].

Parameters	Value %
SiO_2_	>96
Al_2_O_3_	<2.0
Fe_2_O_3_	<1.0
TiO_2_	<0.1

**Table 2:** Result of the sieve testing [37].

Mesh Size (mm)	Residue on the Sieve	
	Scope	(%)
2	Max. 10	8
1.6	10–20	15
1	30–60	50
0.8	10–20	12
0.5	10–20	10
R	Max. 10	5

**Table 3:** Parameters determined on the test stand.

Variable	Symbol	Unit	Value
Speed-length ratio	*v*	m/s	0.4954
Total friction path	*s*	km	5.0527
Time of the test	*t*	min	170

**Table 4:** The chemical composition of the steel provided by the manufacturer [38].

Element Content (%)					
C	Mn	Si	P	S	N
0.2	1.4	-	0.045	0.045	0.009

**Table 5:** Chemical composition of the electrodes with manufacturer’s data [39].

Electrode Number	Chemical Composition (%)						
	C	Mn	Si	Cr	Mo	P	S
Electrode 1	2.1	1.1	0.75	6.5	0.4	-	-
Electrode 2	0.5	0.4	1.8	9	-	-	-
Electrode 3	0.55	0.5	1.5	4.5	0.5	-	-

**Table 6:** Chemical composition of electrodes %.

Element	Electrode 1	Electrode 2	Electrode 3
	Av	Av	Av
C	1.7645	0.3526	0.4728
Si	1.3167	1.3043	1.2554
Mn	1.2791	0.4216	0.5703
P	0.0175	0.0135	0.0285
S	0.0134	0.0108	0.0102
Cr	5.3347	7.1937	4.0569
Mo	0.2944	<0.001	0.393
Ni	0.0587	0.0731	0.0352
Al	0.02	0.0172	0.0083
Cu	0.0764	0.0556	0.0316
B	<0.0005	<0.0005	<0.0005
Nb	<0.001	0.0051	0.0242
Co	0.0103	0.0091	0.0073
V	0.0569	0.0083	0.0545
W	<0.003	<0.003	0.4397
Sn	0.0086	-	0.0082
Pb	<0.003	<0.02	<0.003
As	<0.005	-	<0.005
Bi	<0.003	-	<0.003
Ca	0.0007	-	<0.0005
Ti	0.0092	0.0243	0.0474
Sb	0.0926	-	0.0981
Zn	<0.001	-	<0.001
Zr	0.5252	-	0.0035
Fe	89.117	90.511	92.448
N	-	<0.02	-

**Table 7:** Structure of base sample.

Test Place	Structure Image	Structure Description
Core	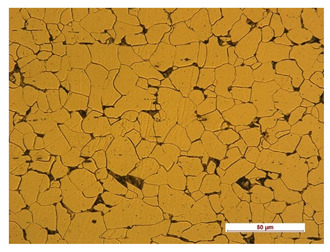	A mixture of ferrite with particles of pearlite

**Table 8:** Structure of the sample—Electrode 1.

Test Place	Structure Image	Structure Description
Padding weld	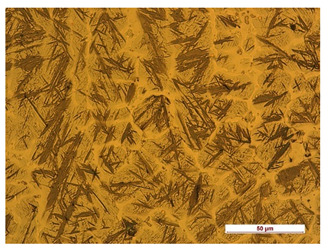	Acicular structure
Weld interface	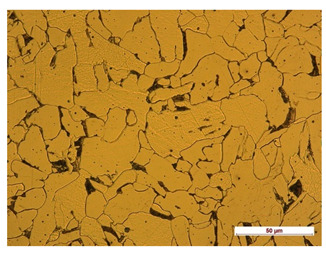	A mixture of ferrite with pearlite precipitations, visible slight overheating of the structure

**Table 9:** Structure of the sample—Electrode 2.

Test Place	Structure Image	Structure Description
Padding weld	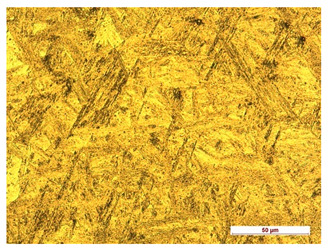	Acicular structure
Weld interface	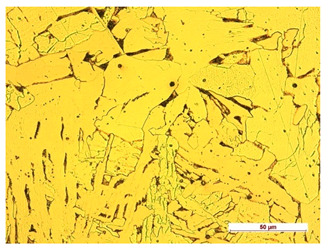	Visible overheating of the structure

**Table 10:** Structure of the sample—Electrode 3.

Test Place	Structure Image	Structure Description
Padding weld	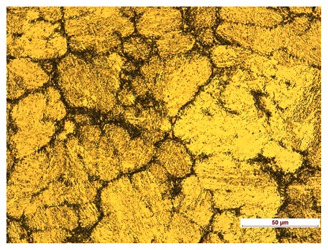	Eutectoid mixture with secondary cementite
Weld interface	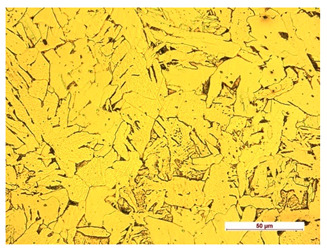	Visible overheating of the structure

**Table 11:** HV_0.1_ microhardness values for samples used in wear tests.

Parameter	Base Sample	Electrode 1		Electrode 2		Electrode 3	
	Base Value	Padding Weld	Weld Interface	Padding Weld	Weld Interface	Padding Weld	Weld Interface
Mean value	129.5	587.9	177.8	589.9	149.6	688.4	209.5
Standard deviation	6.5	41.1	0.7	29.8	16.3	18.4	9.9

**Table 12:** The results of samples wear for the base sample and for the padding weld with three different electrodes in g.

Sample	Mean Value	Minimum	Maximum	Standard Deviation
Electrode 1	0.305	0.204	0.418	0.0694
Electrode 2	0.279	0.243	0.302	0.0227
Electrode 3	0.261	0.220	0.288	0.0262
Base sample	0.342	0.307	0.383	0.0289

**Table 13:** The results of the surface scan of the samples.

Sample	View of the Sample Surface Before Wear Test	View of the Sample Surface after Wear Test
Base sample	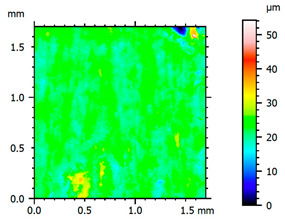	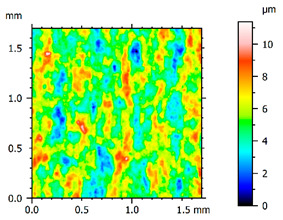
Electrode 1	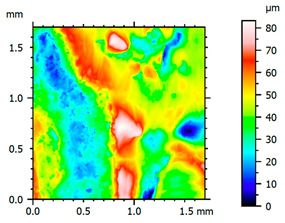	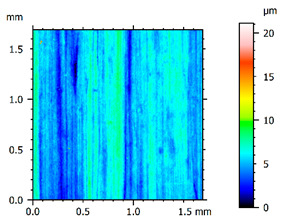
Electrode 2	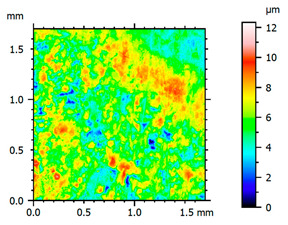	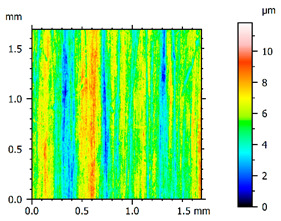
Electrode 3	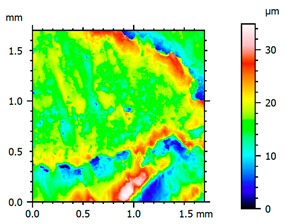	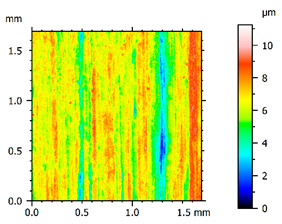

**Table 14:** The basic parameters of the roughness profile of the samples.

Samples		Before Wear Test				After Wear Test			
Parameter	Unit	Base Sample	Electrode 1	Electrode 2	Electrode 3	Base Sample	Electrode 1	Electrode 2	Electrode 3
Rz	µm	6.79	21.92	9.49	19.38	4.85	2.23	2.75	2.27
Ra	µm	1.02	4.59	1.79	4.79	0.70	0.31	0.42	0.34
Sa	µm	1.49	11.68	1.85	5.94	0.86	0.98	1.06	0.97

**Table 15:** The condition of the top layer after 20 times magnification.

Sample	View of the Surface Layer of the Sample Before Wear Test	View of the Surface Layer of the Sample after Wear Test
Base sample	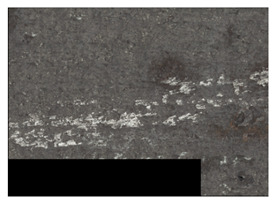	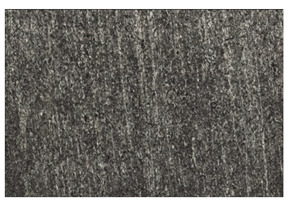
Electrode 1	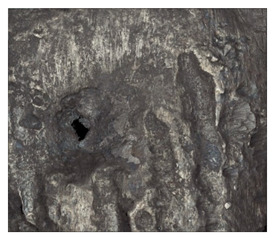	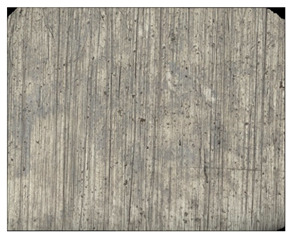
Electrode 2	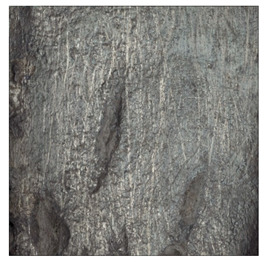	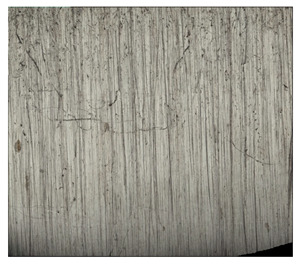
Electrode 3	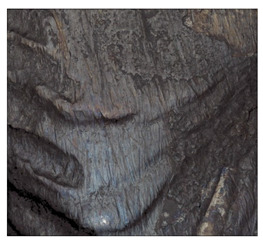	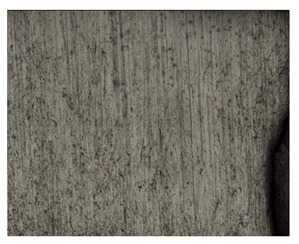

## Data Availability

The data presented in this study are available on request from the corresponding author.
